# The Molecular Characterization and Immunity Identification of *Trichomonas vaginalis* Adhesion Protein 33 (AP33)

**DOI:** 10.3389/fmicb.2020.01433

**Published:** 2020-06-30

**Authors:** Zhenchao Zhang, Yuhua Li, Shuai Wang, Lixia Hao, Yunqing Zhu, Haoran Li, Xiaoxiao Song, Yujuan Duan, Yuhui Sang, Pucheng Wu, Xiangrui Li

**Affiliations:** ^1^Xinxiang Key Laboratory of Pathogenic Biology, School of Basic Medical Sciences, Xinxiang Medical University, Xinxiang, China; ^2^Xinxiang Maternity and Child Health Care Hospital, Xinxiang, China; ^3^MOE Joint International Research Laboratory of Animal Health and Food Safety, College of Veterinary Medicine, Nanjing Agricultural University, Nanjing, China

**Keywords:** *T. vaginalis*, adhesion protein 33, molecular characterization, animal challenge, immunogenicity

## Abstract

Trichomoniasis is caused by *Trichomonas vaginalis* (*T. vaginalis*), which is a widespread and serious sexually transmitted pathogen in humans. The procedure of *T. vaginalis* adherence to the host cell is the precondition for *T. vaginalis* parasitism and pathogenicity. The AP33 adhesin of *T. vaginalis* (TvAP33) plays a key role in the process of adhesion. In this study, the specific primers for polymerase chain reaction (PCR) were designed based on the sequence of TvAP33 (GenBank Accession No. U87098.1) to amplify the open reading frame (ORF), and the ORF was inserted into pET-32a (+) to produce recombinant TvAP33 (rTvAP33). The sequence analysis indicated that the TvAP33 gene encoded a protein of 309 amino acids with 32.53 kDa, and the protein was predicted to have a high antigen index. Western blotting assay showed rTvAP33 was successfully recognized by the sera of mice experimentally infected with *T. vaginalis*, while native TvAP33 in the somatic extract of *T. vaginalis* trophozoite was as well detected by sera from rats immunized with the rTvAP33. Immunofluorescence analysis using an antibody against rTvAP33 demonstrated that the protein was expressed and located on the surface of *T. vaginalis* trophozoites. The recombinant protein was emulsified in Freund’s adjuvant and used to immunize BALB/C mice three times at days 0, 14, and 28. The result of animal challenge experiments revealed the levels of IgG, IgG1, and IgG2a, and IL-4, IL-10, and IL17 among rTvAP33 vaccinated animals were integrally increased. Moreover, the rTvAP33 vaccinated animals were apparently prolonged survival time (26.45 ± 4.10) after challenge infection with this parasite. All these results indicated that TvAP33 could be used as vaccine candidate antigen to induce cell-mediated and humoral immunity.

## Introduction

*Trichomonas vaginalis* is a widespread and serious sexually transmitted pathogen in humans, which causes a common infection of the urogenital system. The World Health Organization (WHO) estimated that 276 million people were infected with *T. vaginalis* on a global scale in 2008, increasing by 11% compared to 2005 (World Health Organization, 2012; Jarrett et al., 2019). In the United States, almost 5 million people are infected with *T. vaginalis* every year, and in Japan the infection rate in women is 24.3%. Moreover, the prevalence in target populations in rural Uganda and South Africa was 23.8 and 18.0%, respectively, (Zhang et al., 2018a). *T. vaginalis* can cause atypical pelvic inflammation in women, urethritis and prostatitis in men, as well as trichomonas pneumonia, bronchitis and oral lesions in newborns (Patel et al., 2018). Moreover, premature rupture of membranes, premature delivery, abortion and low birth weight may occur in pregnant women infected with *T. vaginalis*. In recent years, studies have shown that cervical cancer in women, prostate cancer in men and infertility are associated with the infection of *T. vaginalis* (Langston et al., 2019). In addition, the widespread prevalence of *T. vaginalis* increases the risk of human infection with human immunodeficiency virus (HIV) and mycoplasma (Fiori et al., 2013; Makarova et al., 2017).

At present, the prevention and treatment of trichomoniasis is based on drugs, and metronidazole is commonly used in clinical treatment (Graves et al., 2019; Vargas Rigo et al., 2019). However, an increasing number of reports confirm that there are serious problems the generation of drug-resistant strains and mutagenicity of metronidazole, and other drugs against *T. vaginalis* have their own shortcomings (Bitencourt et al., 2018). Therefore, considerable effort has been made to research new anti-*T. vaginalis* drugs. Until now, few drugs with high biological activity and low cytotoxicity have been found that can completely kill and eliminate *T. vaginalis*.

Vaccine inoculation is a good way to control infectious diseases. However, to date, there is no commercialized vaccine against trichomoniasis (Mirasol-Melendez et al., 2018; Mendoza-Oliveros et al., 2019). Therefore, it is of great importance to identify effective and low toxicity alternative drugs and new vaccines against this parasite. The discovery of drug targets and candidate antigens is a prerequisite for exploring alternative drugs and new vaccines against *T. vaginalis*. Furthermore, much research has indicated that DNA vaccines or recombinant antigens can induce both humoral and cell-mediated immune responses (Zhang et al., 2018c; Mavi et al., 2019; Zhang et al., 2019).

Trophozoites of *T. vaginalis* adhesion to host cells, an early and critical step to colonization and infection, is an intensely specific process that is mediated by the adhesion proteins including AP120, AP65, AP51, AP33, and AP23 (Ardalan et al., 2009; Nievas et al., 2018; Phukan et al., 2018). The proteins of AP65 could be secreted out of trophozoite of *T. vaginalis* and attach to the surface of trophozoites and host cells (Garcia and Alderete, 2007). However, the location of AP33 in trophozoites is not clear. These results using both antisense inhibition of gene expression and AP33 synthesis and the heterologous expression of AP33 in *T. foetus* confirmed a role for this protein as an adhesin in *T. vaginalis* (Mundodi et al., 2007). Further study showed that there were two binding regions in AP33 protein, located at the N-terminal and C-terminal of protein sequence, respectively, (Engbring and Alderete, 1998a). Indirect immunofluorescent antibody test indicated the monoclonal antibody against AP33 could significantly inhibit the adhesion of *T. vaginalis* to HeLa cell (Huang et al., 2007). Moreover, the recombinant AP33 protein showed a high expression level and immunizeed rabbits to produce high titer antibodies, and antibody against AP33 was detected in 78% of the 50 patients infected with *T. vaginalis* by ELISA.

In addition, among the different genotypes of *T. vaginalis*, there is a high homology (98.2–100%) in the AP33 gene sequence (Yuan and Gao, 2005). Results of database retrieval showed that AP33 had significant identity to the succinyl-CoA synthetase α-subunit of several different organisms and virtually 100% identity to the reported *T. vaginalis* subunit (Engbring and Alderete, 1998b). All of these indicated that AP33 could be used as an excellent vaccine candidate antigen against trichomoniasis.

To date, knowledge concerning the immunogenicity of *T. vaginalis* proteins is still limited. In the current study, the cloning and characterization of TvAP33 was completed and the immunogenicity of recombinant TvAP33 was verified in animal experiments.

## Materials and Methods

### Ethics Statement

The experiments in this study were conducted following the guidelines of the Animal Ethics Committee, Xinxiang Medical University, Henan, China (Reference No. 2015016). All efforts were made to alleviate the distress and pain of the experimental animals as much as possible. In this research, euthanasia was used at humane endpoints. Generally, euthanasia was conducted by placing the animals in a closed space and exposing them to 60–70% CO_2_ for 5 min. Occasionally, cervical dislocation was also used for confirmation of valid euthanasia.

### Mice and Parasites

Six-week-old female BALB/c mice were provided by Beijing Vital River Laboratory Animal Technology Co., Ltd. (Beijing, China) and kept under SPF condition (specific pathogen free).

In this study, the strain of *T. vaginalis* was isolated from the vaginal secretions of women with some clinical symptoms of trichomoniasis at the Third Affiliated Hospital of Xinxiang Medical University. *T. vaginalis* were cultured on TYM medium with antibiotics (50 mg/ml ciprofloxacin, 100 mg/ml ceftriaxone), fungicide (2.5 mg/ml amphotericin B) and 10% calf serum in a humidified chamber containing 5% CO_2_ at 37°C. We harvested parasites at the stationary phase by centrifugation (2 × 10^6^ parasites) and used them in subsequent research. The strain of *T. vaginalis* was identified as actin genotype E by PCR-restriction fragment length polymorphism (PCR-RFLP), which was the dominant genotype in the city of Xinxiang, Henan Province, China.

### Soluble Trophozoite Antigens of *T. vaginalis*

A total of 5 × 10^7^ *T. vaginalis* trophozoite cells were harvested from TYM complete medium, centrifuged three times at 2500 rpm for 10 min using 0.1 M PBS (pH 7.2) to wash the cells. After resuspending the obtained pellet in 2 ml PBS, the obtained solution was subjected to repeated freeze-thaw cycles three times at temperatures below −20°C and 4°C to break the cell membrane and purify the protein. Afterward, the lysed cell mixture was treated ultrasonically on ice at a speed of 60 W/s and then separated by centrifugation at 12000 rpm and 4°C for 30 min, after which the supernatant containing protein was obtained. The concentration of the protein samples was determined by the Bradford method, and bovine serum albumin (BSA) was used as the standard. The proteins were filtered sterilized, and finally, the obtained samples of *T. vaginalis* were aliquoted and stored at −70°C for subsequent use.

### Total RNA Extraction of *T. vaginalis*

The extraction of total RNA from *T. vaginalis* trophozoites was conducted by using the E.Z.N.A.^TM^ Total RNA Kit I (OMEGA, Zhengzhou, China) in strict accordance with the manufacturer’s instructions. Water treated with diethyl pyrocarbonate (DEPC) and supplemented with ribonuclease inhibitor (TaKaRa, Dalian, China) was used to resuspend the RNA samples and RNase-free DNase I (TaKaRa) was used to treat the samples before conducting reverse transcription to avoid contamination from genomic DNA. The OD260 was measured to quantify the RNA samples, and the ratio of OD260/OD280 was used to determine its quality. RNA with OD260/OD280 values ranging from 1.9 to 2.0 was considered acceptable.

### Amplification of the ORF of TvAP33

cDNA was amplified by performing RT-PCR. Primers containing protective bases (italics) and restriction enzymes (underlined) (*Bam*H I anchored forward primer, 5′- *CGC*GGATCCATGCTCTCCTCTTCCTTCGAGC-3′; *Xho* I anchored reverse primer, 5′- *CCG*CTCGAGTTAGATCTTGCCCATTCTCTTCATC −3′) were used in PCR to amplify the entire ORF of TvAP33 (GenBank accession no. U87098.1) from trophozoite cDNA. The ORF of TvAP33 amplified by PCR was cloned into the pMD19-T vector (TaKaRa) and transformed into *E. coli* (DH5a) competent cells (Yi Fei Xue Biotechnology, Nanjing, China). The recombinant pMD19-T-TvAP33 clone was amplified by PCR and digested for identification, and 3 positive clones were sequenced for further confirmation. The online tool BLAST^1^ was used to analyze the sequence identity of the fragment inserted in the recombined plasmid using the GenBank database.

### Sequence Analysis

The similarity between sequences was evaluated by BLASTX and BLASTP^2^. CLUSTALW1.8 was used to align the sequences of adhesion proteins. The following online tools were applied for the prediction of motifs, secondary structures and signal peptides: Motifscan^3^, PSIpred^4^, GPI Modification Site Prediction^5^, TMHMM^6^ and SignalP^7^.

### Expressing and Purifying the Proteins TvAP33 and pET-32a

According to the method described in the reported literature (Engbring and Alderete, 1998b; Zhang et al., 2020), the protein of TvAP33 was expressed and purified. The Bradford procedure (Bradford, 1976) was applied to determine the TvAP33 protein concentration with the standard of bovine serum albumin (BSA) used as a standard. The obtained protein was stored at −20°C for further usage.

Similarly, *E. coli* BL21 cells after transformation with the pET-32a (+) plasmid were induced to obtain the pET-32a protein with 6 histidines and TrxTag**^TM^** thioredoxin protein containing 109 amino acid residues.

### Antisera Against Recombined TvAP33 and *T. vaginalis*

SD rats purchased from Beijing Vital River Laboratory Animal Technology Co., Ltd. (Beijing, China) were injected subcutaneously into multiple sites with a mixture of Freund’s complete adjuvant and 0.3 mg pure recombined TvAP33 protein (ratio 1:1) to generate a polyclonal antibody. Fourteen days later, the rats were administered one booster injection containing the mixture of Freund’s incomplete adjuvant and the same antigen (ratio 1:1) and were reboosted three times at an interval of 7 days. After the entire immune procedure was completed, sera were obtained and stored for further use. Negative control serum was obtained before injection (Yanming et al., 2007).

Mice experimentally injected with *T. vaginalis* were used to collect antiserum against *T. vaginalis* (mouse antisera) 10 days post-infection.

### Immunoblot Analyses of Natural and Recombinant TvAP33

SDS–PAGE was used to separate the samples that contained recombinant TvAP33 and soluble trophozoite antigens of *T. vaginalis*, and a nitrocellulose membrane (Millipore, Shanghai, China) was used for protein hybridization after separating the proteins in the gel. The membranes were blocked with TBST containing Tween 20, Tris-buffer saline and 5% (w/v) skim milk powder and then incubated with mouse antiserum (1:100) or rat antiserum (1:200) as the primary antibodies for 1 h at 37°C. Then, TBST was used to wash the membranes three times, and horseradish peroxidase (HRP)-conjugated goat anti-mouse IgG and HRP-conjugated goat anti-rat IgG (Sigma, Shanghai, China) were added to the membranes for 1 h at 37°C. Finally, a 3, 3′-diaminobenzidine tetrahydrochloride (DAB) kit (Boster Bio-Technology, Wuhan, China) was used to detect the bands strictly following the manufacturer’s instructions.

### Expression and Location of TvAP33 in Trophozoites of *T. vaginalis* by Immunofluorescence

Harvested *T. vaginalis* trophozoite cells were washed three times with PBS (pH 7.2), and smeared on a poly-L-lysine treated glass slide for 15 min. Then, the trophozoites were fixed using PBS containing 4% paraformaldehyde for 10 min at room temperature, permeabilized using PBS containing 1% TritonX-100 for 10 min, washed three times with PBS and blocked using PBST containing 4% (w/v) BSA for 1 h at 37°C. After the slides were washed three times with PBS, rat antiserum against TvAP33 (dilution ratio 1:100) and control rat serum were added to the slides overnight at 4°C. The slides were washed three times with PBS, treated with goat anti-rat IgG antibody (Beyotime, Shanghai, China) labeled with Cy3 (dilution ratio 1:1,000) and incubated in the dark for 40 min. After three washes in PBS, DAPI (Beyotime) was used to stain the nuclei for 15 min in darkness. After washing with PBS, fluorescent mounting medium (Beyotime) was added, and the cells were examined by laser confocal microscopy (Nikon, Beijing, China).

### Immunization and Challenge Infection

In total, 80 BALB/c mice aged 6 weeks old were classified at random into four groups (20/group) and injected subcutaneously with 100 μg of recombinant TvAP33 protein and Freund adjuvant (1:1), pET-32a protein mixed with Freund adjuvant (1:1), or the same volume of Freund adjuvant alone. The remaining group of mice was used as the blank control and received no inoculation. The animals in all groups were vaccinated three times at 0 day (Freund’s complete adjuvant was used), 14 day (Freund’s incomplete adjuvant was used) and 28 day (Freund’s incomplete adjuvant was used) separately. Ten days after the last vaccination, each mouse in the experiment was challenged with 1 × 10^7^ trophozoites of *T. vaginalis* intraperitoneally, and the survival of mice was monitored throughout the month after challenge. Animals showing symptoms were subjected to euthanasia by CO_2_.

Thirty days later, the following formula was used to calculate the survival rate of mice challenged with *T. vaginalis*: the number of surviving mice/the number of mice before immunization × 100%.

### The Use of Enzyme-Linked Immunosorbent Assay (ELISA) to Determine Antibody Levels in Sera

The collection of mouse blood samples in each group (*n* = 5) was conducted at 0, 14, 28, and 42 days, and the obtained serum samples were stored at −20°C in order to further evaluate antibodies and measure cytokines. Indirect ELISA was performed to detect IgG isotypes and specific anti-TvAP33 antibodies as described in a previous study with a few modifications (Zhang et al., 2018c). In brief, microtiter plates (Costar, New York, NY, United States) were coated with recombinant TvAP33 in carbonate buffer (2.5 μg/ml, 100 μl/well) with a PH value of 9.6 overnight at 4°C and blocked with 4% BSA for 2 h at 37°C. A mouse serum dilution (ratio = 1/10, PBS was used as the diluent) was added to the wells, and the plates were incubated at 37°C for 2 h. After the plates were washed three times with PBST, the plates were treated with the HRP-conjugated secondary antibodies goat anti-mouse IgG2a, IgG1, and IgG (SouthernBiotech, Birmingham, AL, United States). Finally, 100 μl of 3,3,5,5-tetramethylbenzidine was added into each well, and then 100 μl (2 M) sulfuric acid was added to terminate the reaction. The light absorption at 450 nm was measured by an automatic ELISA reader (MULTISKAN FC, Thermo Fisher Scientific, Waltham, MA, United States) and all tests were completed in triplicate.

### Cytokine Determination

The cytokine expression levels were determined using serum samples from all experimental rodents. Commercial ELISA kits (Boster, Wuhan, China) were used to measure IFN-γ, interleukin-2 (IL-2), interleukin-4 (IL-4), interleukin-10 (IL-10), and interleukin-17 (IL-17). Recombinant IFN-γ, IL-17, IL-10, IL-4, and IL-2 were used to generate the corresponding standard curves based on which the cytokines were quantified. The analyses were conducted based on data obtained from 3 individual experiments.

### Statistical Analyses

The SPSS statistical package (SPSS for Windows 16; SPSS Inc., Chicago, IL, United States) was utilized to apply Duncan’s multiple range test and one-way analysis of variance (ANOVA) to determine the statistical significance of biological research data, such as the expression of cytokines and corresponding antibody levels. The survival periods were compared using the Kaplan–Meier method. Tests of the differences among groups were conducted, and the threshold value of *p* < 0.05 indicated that the difference was statistically significant.

## Results

### Cloning and Sequence Analysis of TvAP33

**FIGURE 1:**
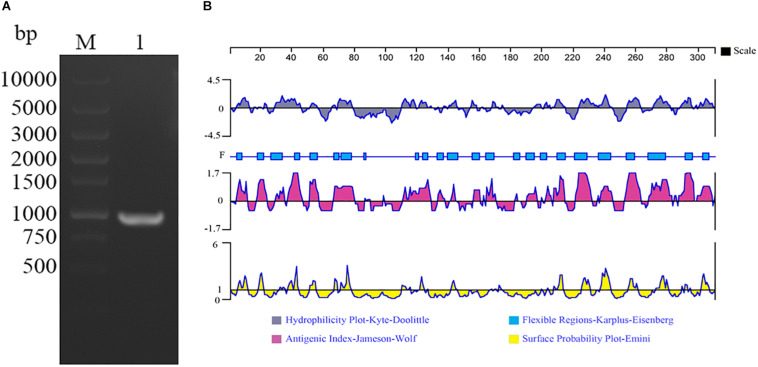
Amplification and bioinformatics analysis of the TvAP33 gene. **(A)** Agarose gel electrophoresis of the TvAP33 ORF. (Lane M) DNA molecular weight marker DL 10000 (values are in bp); (Lane 1) the ORF of TvAP33. **(B)** The linear-B cell epitopes of TvAP33 predicted by DNASTAR in terms of hydrophilicity plot, flexible regions, antigenic index, and surface probability rules.

A 930 bp ORF of TvAP33, which encodes a protein of 309 amino acids with a molecular mass of 32.53 kDa, was found that started from the ATG initiation codon and ended at the stop codon of TAA, as demonstrated by gel electrophoresis (Figure 1A) and sequencing. By sequence analysis, it was discovered that the ORF of TvAP33 contained 35 strongly basic amino acid residues, 24 strongly acidic amino acid residues, 113 hydrophobic amino acid residues and 62 polar amino acid residues with a theoretical pI of 9.47. In comparison to known protein and nucleotide sequences recorded by the NCBI database^2^, the TvAP33 nucleotide sequence was 99% identical to the *T. vaginalis* succinyl CoA synthetase-3 alpha-subunit (L31931.1) and hypothetical protein (XM_001328094.1) sequence. The TvAP33 amino acids sequence exhibited 99% homology to the hypothetical protein of *T. vaginalis* (XP_001328129.1) sequence and 97% homology to the Succinyl-CoA synthetase subunit alpha-1 of *T. vaginalis* (P53399.1) sequence in NCBI. In the predicted protein, one N-glycosylation site and nineteen phosphorylation sites were predicted, but no transmembrane domains, GPI anchors or signal peptides were discovered. As shown in Figure 1B, the protein had seven hydrophilic regions, 4—12, 29—46, 65—78, 223—245, 254—262, 271—281 and 288—309, and nine highly antigenic indices and consecutive regions, 4—12, 17—23, 29—45, 51—56, 66—79, 111—128, 190—213, 221—244 and 253—308, and many regions of TvAP33 were flexible regions. The protein also contained three Succinyl-CoA synthetase regions, one CoA binding site, one CoA-ligase domain, and one NAD(P) binding domain of glutamate dehydrogenase. μm

### Expressing and Purifying Recombinant TvAP33

**FIGURE 2:**
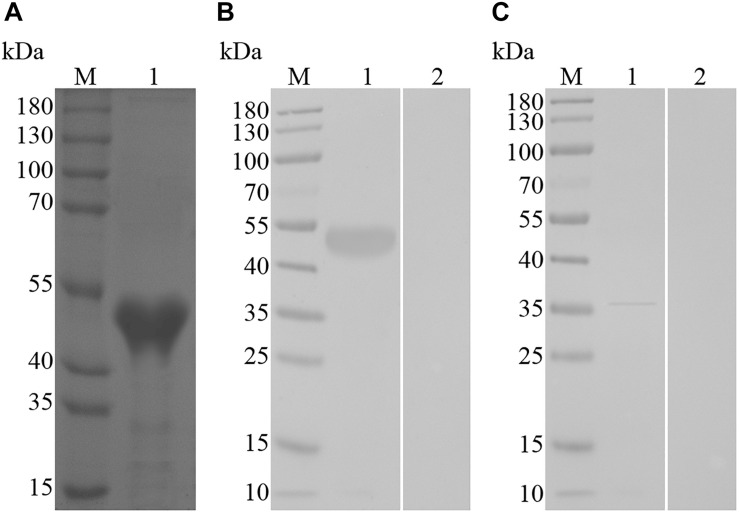
**(A)** SDS–PAGE of purified recombinant TvAP33 protein. (Lane M) Protein mark (values are in kDa); (Lane 1) recombinant TvAP33 protein. **(B)** Immunoblot for recombinant TvAP33. (Lane M) Protein mark (values are in kDa); (Lane 1) recombinant TvAP33 protein probed with serum from mice experimentally infected with *T. vaginalis* as the primary antibody; (Lane 2) recombinant TvAP33 protein probed with serum of normal mice as the primary antibody. **(C)** Immunoblot of crude somatic extracts of *T. vaginalis* trophozoites. (Lane M) Protein mark (values are in kDa); (Lane 1) crude somatic extracts of *T. vaginalis* trophozoites probed with serum from rats immunized with the TvAP33 protein; (Lane 2) crude somatic extracts of *T. vaginalis* trophozoites probed with serum of normal rats without immunization as the primary antibody.

The SDS–PAGE result revealed that the supernatant of bacterial sonication extraction exhibited the highest levels of recombinant TvAP33, which was subsequently purified from the supernatant using Ni-NTA chromatography and isolated by SDS–PAGE gel as an individual band with a molecular weight of 51 kDa (Figure 2A). The molecular mass of the recombinant protein was deduced to be 32.53 kDa after subtracting the fused protein of 18 kDa.

### Analyzing the Native and Recombinant TvAP33 Using Immunoblot

The immunoblot results revealed that the recombinant TvAP33 protein was recognized by serum from mice subjected to artificial *T. vaginalis* infection instead of serum from healthy mice (Figure 2B). The western blot results also revealed the native TvAP33 protein was recognized by rat anti-TvAP33 serum, which was a band with a molecular mass of approximately 36 kDa in the somatic extraction of *T. vaginalis* trophozoites (Figure 2C) and slightly larger than the predicted protein.

### Expression and Location of TvAP33 in Trophozoites of *T. vaginalis*

**FIGURE 3:**
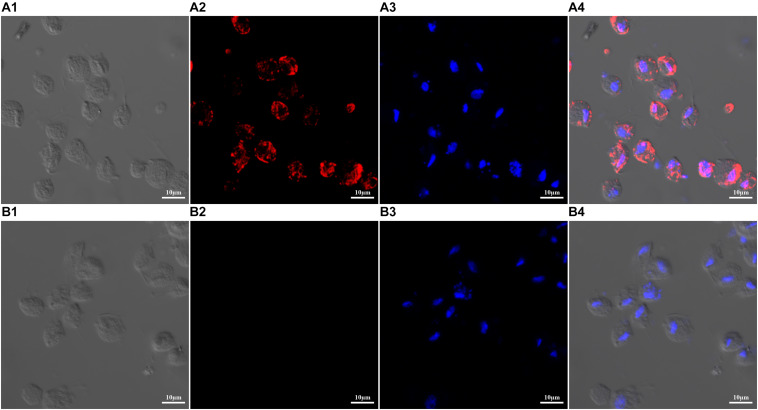
Expression and localization of TvAP33 protein in *T. vaginalis* trophozoites by immunofluorescence assay (×40 magnification). **(A)** *T. vaginalis* trophozoites were probed with serum from rats immunized with TvAP33 protein. **(A1)** Differential interference contrast (DIC). **(A2)** Immunofluorescence localization using Cy3. **(A3)** Nuclei were stained with DAPI. **(A4)** DIC, Cy3 and DAPI merged. **(B)** Negative control, trophozoites were probed with serum from normal rats without immunization as the primary antibody. **(B1)** DIC. **(B2)** Cy3. **(B3)** DAPI. **(B4)** Merged.

The expression and location of TvAP33 protein was investigated in trophozoites (Figure 3) with anti-rTvAP33 serum. It was revealed that the trophozoites were stained by the fluorescent label, which was absent from the slides in the negative control group, and the location of TvAP33 was mainly on the surface of trophozoites.

### Evaluating the Protective Effect of Inoculation on Experimental Rodents Against Pathogen Challenge

**FIGURE 4:**
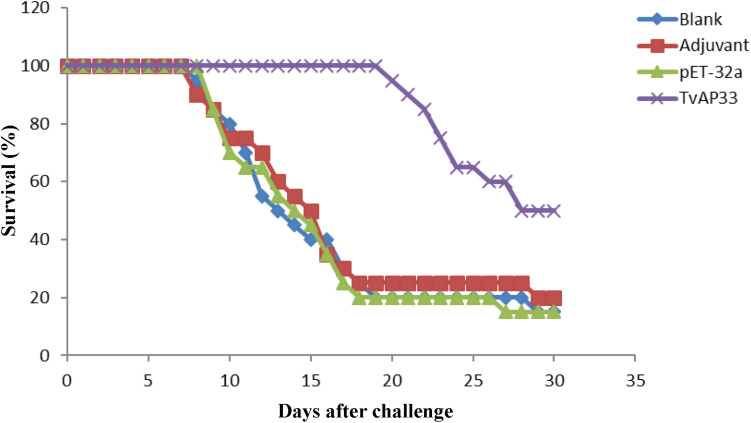
Survival curve of mice after challenge infection with *T. vaginalis* trophozoite actin genotype E strain. Mice were challenged with 10^7^ trophozoites of the *T. vaginalis* actin genotype E strain intraperitoneally 10 days after the last vaccination.

BALB/c mice were used to investigate the protection of the recombinant protein as a vaccine antigen against artificial infection with *T. vaginalis*. The mice were subjected to one primary immunization and two booster immunizations (Table 1) and then injected intraperitoneally with 1 × 10^7^ *T. vaginalis* trophozoites. The survival rate of the different groups of mice challenged with *T. vaginalis* is presented in Figure 4. The survival rate of the mice in the TvAP33 group was significantly higher than that of the blank, Freund adjuvant and pET-32a protein groups. In the blank, Freund adjuvant and pET-32a protein groups, most mice (75–80%) died within 18 days. Moreover, the survival time of mice subjected to TvAP33 injection (26.45 ± 4.10, *P* < 0.05) was significantly longer than that of the mice injected with Freund adjuvant or pET-32a protein.

**TABLE 1:** Immunization protocol in experimental and control groups.

**Groups**	**1st (0 Day)**	**2nd (14 Days)**	**3rd (28 Days)**
Blank control	No immunity	No immunity	No immunity
Adjuvant control (μl)	200	200	200
pET-32a protein control (μg)	100	100	100
Recombinant TvAP33 protein (μg)	100	100	100

### Humoral Immunoreactions

**FIGURE 5:**
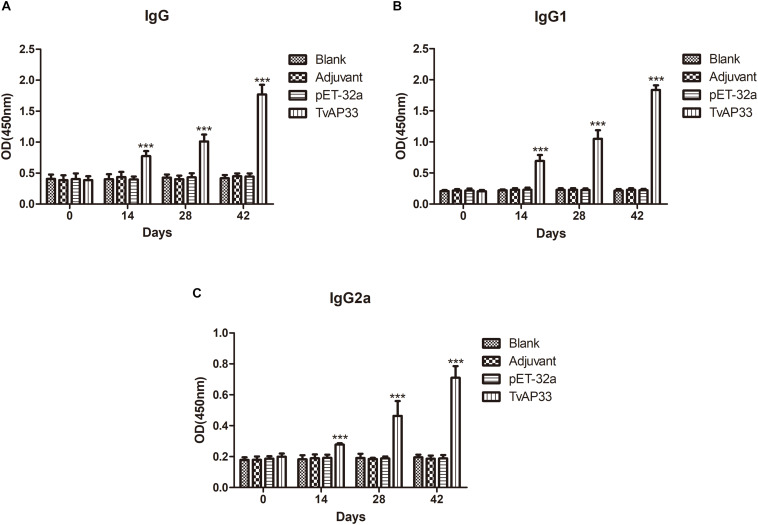
The dynamics of humoral response in BALB/c mice induced by recombinant TvAP33 protein. The BALB/c mice were randomly divided into four groups of five mice each (*n* = 5). The BALB/c mice in group were immunized with recombinant protein TvAP33 mixed with Freund adjuvant (1:1), pET-32a protein mixed with Freund adjuvant (1:1), or Freund adjuvant alone, and the rest of the group as a blank control. The titers of IgG and the subclasses IgG1 and IgG2a were detected on days 0, 14, 28, and 42. The results were expressed as mean ± SD with respect to absorbance at 450 nm. Statistically significant differences (*P* < 0.05), (*P* < 0.01), and (*P* < 0.001) were indicated by (*), (**), and (***) in different groups at the same time point, respectively. **(A)** IgG. **(B)** IgG1. **(C)** IgG2a.

To evaluate the different antibody responses induced by the 3 sequential vaccinations, the distribution pattern of IgG2a and IgG1 isotypes as well as the IgG level were examined each time after injection. In comparison to those in the control group, the IgG levels in serum of mice injected with TvAP33 were significantly higher (*P* < 0.001). In addition, the OD value of IgG continually increased when the animals were injected with TvAP33, and the IgG titers peaked after at 3rd immunization. No significant differences in IgG levels were discovered among the control groups (Figure 5A). In comparison to those in mice in the control groups, the levels of IgG1 and IgG2a in mice subjected to TvAP33 injection were among the highest (*P* < 0.001; Figures 5B,C), and IgG2a levels were obviously less than IgG1, suggesting the induction of Th2-type cellular immunity by TvAP33.

### Cytokine Levels in Sera of Immunized Mice

**FIGURE 6:**
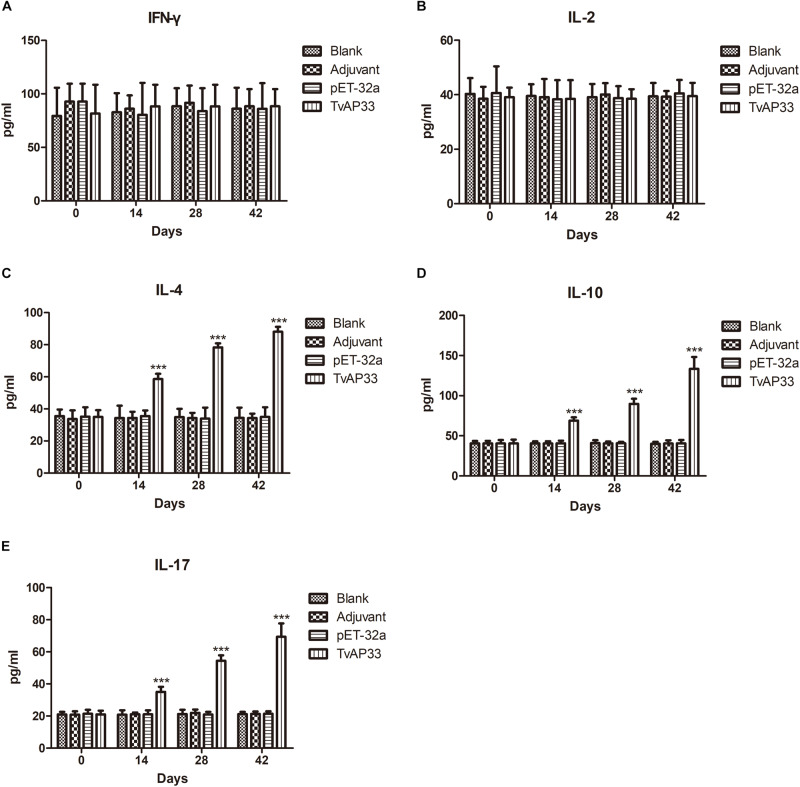
Cytokine production. The BALB/c mice were randomly divided into four groups of five mice each (*n* = 5). The BALB/c mice in the appropriate groups were immunized with recombinant protein TvAP33 mixed with Freund adjuvant (1:1), pET-32a protein mixed with Freund adjuvant (1:1), Freund adjuvant alone and the last group as blank controls. The levels of cytokines were determined by antigen-captured ELISA at days 0, 14, 28, and 42, and the comparison results are expressed as mean ± SD of pg/ml. Statistically significant differences (*P* < 0.05), (*P* < 0.01), and (*P* < 0.001) are indicated by (*), (**), and (***) in different groups at the same time point. **(A)** IFN-γ. **(B)** IL-2. **(C)** IL-4. **(D)** IL-10. **(E)** IL-17.

As shown in Figure 6, serum samples collected at days 0, 14, 28, and 42 were used to measure the levels of IFN-γ, IL-2, IL-4, IL-10, and IL-17 in the experimental groups. The results indicated that the quantities of IL-4, IL-10, and IL-17 (Figures 6C–E) in mice injected with TvAP33 were significantly higher than those in the control groups on days 14, 28, and 42 after vaccination (*P* < 0.001), and the levels of IL-4, IL-10, and IL-17 were highest throughout the whole experiment after the 3rd immunization. However, the IFN-γ and IL-2 levels of the experimental groups were not significantly different from those of the controls (Figures 6A,B).

## Discussion

The colonization of the urogenital tract of humans by the protozoan parasite *T. vaginalis* is responsible for trichomoniasis (Kassai et al., 1988). With the aim of preventing and controlling contagious diseases, vaccines are normally developed as the method to block pathogens with the most cost effective performance (Peggy et al., 2015; Smith and Garber, 2015). Nevertheless, the most difficult step in developing vaccines is identifying the appropriate candidate antigens. Adherence of the parasite to the vaginal epithelium, a property key to colonization and infection, is a highly specific event that is mediated by the adhesins AP120, AP65, AP51, AP33, and AP23 (Alderete and Garza, 1988; Arroyo et al., 1992). These five proteins reside on the surface of *T. vaginalis*. The association between the amounts of adhesins and the levels of cytoadherence has been demonstrated. There were two binding regions in AP33 protein, which were located at the N-terminal and C-terminal of protein sequence, respectively, (Engbring and Alderete, 1998a). Huang et al. confirmed that the monoclonal antibody against AP33 could significantly inhibit the adhesion of *T. vaginalis* to HeLa cell (Huang et al., 2007). Thus, AP33 played an important role in adhesion to host cells and could be used as candidate vaccine antigen to control trichomoniasis. In our study, molecular properties of TvAP33 were analyzed, and the immunoprotective effect of TvAP33 against *T. vaginalis* was identified. Moreover, the gene sequence of TvAP33 was 983 bp, which contained a 930 bp ORF. The protein encoded by the TvAP33 ORF contains 309 amino acid residues, the molecular weight of which is 32.53 kDa, which are consistent with previous studies (Engbring and Alderete, 1998b). Based on the DNASTAR analysis result of the protein sequence of TvAP33, TvAP33 was determined to have strong antigenicity due to outstanding surface probability and antigenic index resulting from the extensively distributed hydrophilic and flexible regions in the protein.

Alignment between TvAP33 and the available sequences in NCBI databases showed that the nucleotide sequence of TvAP33 had 99% to *T. vaginalis* succinyl CoA synthetase-3 alpha-subunit and the protein sequence of TvAP33 had 97% to the Succinyl-CoA synthetase subunit alpha-1 of *T. vaginalis*. In addition, BLASTX and BLASTP analyses revealed that three Succinyl-CoA synthetase regions, one CoA binding site, one CoA-ligase domain and one NAD(P) binding domain of glutamate dehydrogenase existed in the TvAP33 sequence. Engbring et al. indicated that the AP33 sequences were almost identical to the three reported sequences encoding the *T. vaginalis* α-subunit of Succinyl-CoA synthetase, with two base changes for ap33-1, one base change for ap33-2 and three base changes for ap33-3, resulting in only one amino acid change for AP33-1 (Engbring and Alderete, 1998b). However, whether TvAP33 has the catalytic activity of Succinyl-CoA synthetase and glutamate dehydrogenase in the parasitic process of *T. vaginalis* needs further study.

Additionally, sequence analysis showed that there were almost no similarities in the amino acid epitope sequences between TvAP33 and the human homolog sequences, which would avoid potential autoimmune problems in future applications. Overall, the expression of TvAP33 as a functional protein was sustained throughout the whole life cycle of *T. vaginalis*, especially in the process of adherence to host cells (Garcia and Alderete, 2007). And the recombinant AP33 protein showed a high expression level and immunized rabbits to produce high titer antibodies, and antibody against AP33 was detected in patients infected with *T. vaginalis* by ELISA (Liang et al., 2006). Consequently, TvAP33 is qualifies as an ideal candidate for vaccine development due to the above advantages.

We found that a band with a molecular weight of 36 kDa in the somatically extracted *T. vaginalis* trophozoites was recognized by the serum raised against recombinant TvAP33. The molecular weight of native TvAP33 was slightly larger than that of the predicted protein, which was 32.53 kDa. The TvAP33 protein might be post-translationally modified since it possesses phosphorylation and glycosylation sites according to the sequence analyses.

According to the results of western blots, serum samples from mice subjected to experimental infection of *T. vaginalis* trophozoites recognized the recombinant TvAP33, indicating that the immune system was able to recognize TvAP33 which subsequently induced an antibody response.

Protein localization studies in parasites helps to understand the function of localized proteins and adhesion proteins, based on which TvAP33 is normally considered to be involved in cellular adhesion. In the current study, it was verified that TvAP33 was located mainly at the surface of trophozoites. Previous study showed that two domains interactive with host cell surfaces were identified at distinct parts of AP33: one in the N-terminal half of the protein, and the other within 24 residues in the C-terminal third (Engbring and Alderete, 1998a). So, the protein is involved in the attachment of the parasite to the host cell during the contact process between host cells and parasites. Whether TvAP33 adheres to the host cell surface in a ligand-receptor manner on the trophozoites of the *T. vaginalis* surface needs additional studies. However, sequence analysis showed that no transmembrane domains or signal peptides were discovered in TvAP33. How TvAP33 is localized on the parasite surface needs further study.

Cytokines play an essential role in activating Th cells (Paintlia et al., 2002). Known as an inflammatory factor, IFN-γ, defends against infections by pathogens and activates Th1 cells (Xu et al., 2016). IL-2 promotes the differentiation of T cells into effector T cells and into memory T cells when the initial T cell is also stimulated by an antigen, thus helping to fight off infections (Liao et al., 2011). In addition, IFN-γ is often considered as a marker of Th1 cells, which express a large amount of IL-2. In this research, it was indicated that TvAP33 could not induce the expression of IFN-γ and IL-2, which indicated that as a vaccine, TvAP33 could not induce a Th1 immune response.

IL-4 promotes the proliferation, differentiation and maturation of B cells and the differentiation of CD4^+^ T cells to Th2 cells, along with antibody expression, and it is considered a cytokine marker of Th2 cells (Isabelle et al., 2013). IL-10 is an important cytokine that plays an essential role in inflammation and the regulation of immunity. Moreover, the expression of Th1 cytokines, MHC class II antigen and costimulatory molecules is downregulated by IL-10 in macrophages (Beebe et al., 2015). The antibody response, proliferation and survival of B cells are enhanced, and the activity of NF-κB is blocked by IL-10 (Wei et al., 2015). In addition, IL-10 participates in regulating the JAK-STAT signaling pathway (Su et al., 2017). Xie et al. reported that the vaccine containing the recombined α-actinin subunit of *T. vaginalis* as the antigen stimulated a significant increase in IL-10 levels (Xie et al., 2017). In this study, the recombinant protein TvAP33 resulted in significantly increased IL-4 and IL-10 expression in mice vaccinated with TvAP33. This proved TvAP33 as vaccine could induce Th2 immune response.

Numerous immune regulatory functions have been reported for the IL-17 family of cytokines, presumably due to their induction of many immune signaling molecules (Li et al., 2015; Ma et al., 2019). The most notable role of IL-17 is its involvement in inducing and mediating proinflammatory responses (van Dalen et al., 2019). IL-17 is commonly associated with allergic responses. IL-17 is expressed by Th17 cells, which are a subset of CD4^+^ cells (Wright et al., 2007). In the current study, TvAP33 was capable of stimulating high IL-17 levels.

The production of antibodies specific to pathogens not only plays an important role in regulating the immune reaction mediated by cells but also suppresses parasites adhering to host cells through receptor molecules (Zhang et al., 2018b). In addition, intracellular parasites are also coated and killed by macrophages, which are recruited by these antibodies (Zhang et al., 2019). In the current study, in comparison to the levels in mice receiving the control vaccine, the anti-*T. vaginalis* IgG levels in mice vaccinated with recombinant TvAP33 were increased. Further analyses of IgG subclasses revealed that the levels of IgG1 were higher than those of IgG2a, suggesting that TvAP33 was capable of inducing a Th2-mediated immune response and thus exerted an important effect on the immunity of hosts against *T. vaginalis*. Thus, TvAP33 could stimulate significant expression of IL-4 and IL-10 (Th2-type cytokine).

At present, efforts have been made to develop vaccines against *T. vaginalis* (Abraham et al., 1996; Smith and Garber, 2015). Among these studies, 2 were focused on producing a vaccine that contained whole *T. vaginalis* cells and was injected subcutaneously into mice that were challenged through the vagina. It has also been reported that intraperitoneal injection of *T. vaginalis* can be used as a mouse model to study vaccines against *T. vaginalis* (Xie et al., 2017). Previous studies indicated that the initial infection efficiency of intraperitoneal injection was higher than that of vaginal infection (Hernández et al., 2010; Xie et al., 2017). In our current study, the effect of TvAP33 against *T. vaginalis* was evaluated by intraperitoneal challenge with 1 × 10^7^ trophozoites.

*In vivo* protection is considered one of the most critical outcomes in assessing the value of a candidate vaccine (Hammond et al., 1990; Peggy et al., 2015). The survival time and rate of the animals that receive vaccination and are challenged with parasites are considered among the most widely accepted ways of evaluating the protection effect of a vaccine. In our study, TvAP33 had a protective effect (50%) that was higher than that of the controls. Moreover, survival assay results revealed a remarkable increase in survival time (26.45 ± 4.10 days) due to TvAP33 vaccination, suggesting that the vaccine was able to induce specific immune responses against *T. vaginalis* infections in BALB/c mice. Nevertheless, whether the immune responses induced by TvAP33 could protect the challenged mice from trichomoniasis and subsequent mortality in a longer follow-up period needs further study.

In conclusion, the cloning of the TvAP33 gene and the expression of the TvAP33 protein were completed. Immunoblots showed that TvAP33 was immunogenic and could stimulate the production of specific antibodies. Immunofluorescence indicated that TvAP33 was located on the surface of the trophozoites of *T. vaginalis*. It was revealed by experiments in challenge animals that recombinant TvAP33 protein was capable of inducing partial protective effects in mice challenged with parasites. Moreover, as a vaccine candidate antigen, TvAP33 stimulated the production of Th2 cell immunity. In summary, the potential of TvAP33 as a novel antigen of *T. vaginalis* has been verified by the abovementioned experimental results, although the precise effect of cell adhesion exerted by *T. vaginalis* is still to be determined in the future.

## Data Availability Statement

The raw data supporting the conclusions of this article will be made available by the authors, without undue reservation, to any qualified researcher.

## Ethics Statement

The animal study was reviewed and approved by the guidelines of the Animal Ethics Committee of the Xinxiang Medical University in Henan, China (Reference No. 2015016), was followed throughout all of the experiments of this study.

## Author Contributions

ZZ and XL conceived and designed the experiments, analyzed the data, and wrote the manuscript. YL and PW performed preparation of soluble trophozoite antigens and construction of the prokaryotic expression vector. HL and YD performed expression and purification of proteins of TvAP33 and pET-32a. LH and XS prepared antisera against recombined TvAP33 and *T. vaginalis*. ZZ and YZ completed immunoblotting and immunofluorescence. YL and SW performed BALB/c mice immunization and challenge. XS and YS performed determination of antibodies by ELISA. XL and SW prepared the figures and tables. All authors read and approved the final manuscript.

## Conflict of Interest

The authors declare that the research was conducted in the absence of any commercial or financial relationships that could be construed as a potential conflict of interest.

## Footnotes

1 http://www.ncbi.nlm.nih.gov/BLAST/

2 https://blast.ncbi.nlm.nih.gov/Blast.cgi

3 https://myhits.sib.swiss/cgi-bin/motif_scan

4 http://bioinf.cs.ucl.ac.uk/psipred/

5 http://mendel.imp.ac.at/sat/gpi/gpi_server.html

6 http://www.cbs.dtu.dk/services/TMHMM/

7 http://www.cbs.dtu.dk/services/SignalP/
